# Simultaneous monitoring of three key neuronal functions in primary neuronal cultures

**DOI:** 10.1016/j.jneumeth.2006.09.012

**Published:** 2007-03-15

**Authors:** Gareth John Owen Evans, Michael Alan Cousin

**Affiliations:** Centre for Integrative Physiology, Hugh Robson Building, George Square, University of Edinburgh, Edinburgh EH8 9XD, UK

**Keywords:** [Ca^2+^]_i_, intracellular free calcium, SV, synaptic vesicle, HCS, high content screening, FM1-43, Calcium, Exocytosis, Apoptosis, Neuron, Synaptic vesicle, High content screening

## Abstract

The coupling of Ca^2+^ influx to synaptic vesicle (SV) recycling in nerve terminals is essential for neurotransmitter release and thus neuronal communication. Both of these parameters have been monitored using fluorescent reporter dyes such as fura-2 and FM1-43 in single central nerve terminals. However, their simultaneous monitoring has been hampered by the proximity of their fluorescence spectra, resulting in significant contamination of their signals by bleedthrough. We have developed an assay that simultaneously monitors both SV recycling and changes in intracellular free Ca^2+^ ([Ca^2+^]_i_) in cultured neurons using the reporter dyes FM4-64 and fura-2AM. By monitoring both fura-2 and FM4-64 emission in the far red range, we were able to visualize functionally independent readouts of both SV recycling and [Ca^2+^]_i_ independent of fluorescence bleedthrough. We were also able to incorporate an assay of cell viability without any fluorescence bleedthrough from either fura-2 or FM4-64 signals, using the dye SYTOX Green. We propose that this assay of three key neuronal functions could be simply translated into a high content screening format for studies investigating small molecule inhibitors of these processes.

## 1 Introduction

Neurotransmitter release is essential for communication between neurones and thus brain function. It is stimulated by influx of extracellular calcium (Ca^2+^) via voltage-dependent Ca^2+^ channels on nerve terminal depolarisation by action potentials (Murthy and De Camilli, 2003). Ca^2+^ influx evokes this release by stimulating the fusion of neurotransmitter-containing synaptic vesicles (SVs) with the nerve terminal plasma membrane. This Ca^2+^ signal (and thus neurotransmitter release) can be modulated by changes in either the location (by removal or insertion of channels in the active zone (Passafaro et al., 1994, 1996; Spafford and Zamponi, 2003)), type (different Ca^2+^ channel subtypes have different activation and inactivation kinetics (Dunlap et al., 1995)) or post-translational modification (Hell et al., 1994) of voltage-dependent Ca^2+^ channels in the nerve terminal.

Because of the tight coupling between Ca^2+^ and exocytosis many laboratories have examined their relationship. This has usually involved monitoring evoked neurotransmitter release and calcium influx in a population of neurones, using either isolated nerve terminals, primary cell culture or slices (Nicholls, 1993). However, individual nerve terminals display a heterogeneity of responses to the same stimulus for both Ca^2+^ influx and exocytosis (Murthy et al., 1997), therefore to obtain a true reflection of the relationship between these two parameters, studies that examine the Ca^2+^ coupling of exocytosis must be conducted within the same nerve terminal. This has previously been achieved using a combination of electrophysiology and fluorescence imaging in large atypical central nerve terminals (Matthews, 1996). However, assays to examine the relationship between Ca^2+^ influx and SV exocytosis in a typical synapse have been much more difficult to perform, since central nerve terminals are not accessible to standard electrophysiological techniques.

Approximately 15 years ago the fluorescent styryl dye FM1-43 was shown to be an excellent reporter of activity-dependent SV recycling in both peripheral and central nerve terminals (Betz and Bewick, 1992; Ryan et al., 1993). FM1-43 and its derivatives are now widely used to examine the extent and kinetics of SV recycling in real time in individual nerve terminals using single cell fluorescence imaging techniques (Cochilla et al., 1999; Cousin and Robinson, 1999). This has allowed the simultaneous monitoring of both SV recycling and Ca^2+^ influx (using dyes such as fura-2) in single nerve terminals (Burrone and Lagnado, 1997; Haller et al., 1998, 1999, 2001; Lagnado et al., 1996; Nunez et al., 2000; Shorte et al., 1995). However, the excitation and emission spectra of these dyes overlap, resulting in significant “bleedthrough” of fluorescence signal from one to the other when traditional filter sets are used (Haller et al., 1999, 2001). SV recycling can also be monitored with red-emitting styryl dyes such as FM4-64 and FM5-95, whose spectra do not overlap with ultraviolet (UV) dyes such as fura-2 (http://www.probes.invitrogen.com/). However, since the excitation and emission of these dyes are at opposite ends of the fluorescence spectrum, real-time simultaneous monitoring of these signals has not been attempted, since their emission has to be monitored using the same dichroic and emission filter sets.

We report here the development of a simple new assay to simultaneously monitor SV recycling and Ca^2+^ influx in real time in individual central nerve terminals. The assay can be used on a standard epifluorescence microscope system since it employs standard UV and red fluorescent dye filter sets. Since we utilized dyes from opposite ends of the fluorescence spectrum, we were able to add a cell death parameter to the assay using a green fluorescent indicator. We propose that this assay could be easily converted into a high content multiplexed assay of neuronal function.

## 2 Materials and methods

### 2.1 Materials

Fura-2AM and fura-2 free pentapotassium salt were from Calbiochem (Nottingham, UK). FM4-64 and SYTOX Green were from Molecular Probes (Eugene, OR). Coverslips were from Raymond Lamb (Eastbourne, UK). Tissue culture plastics were obtained from Griener (Dursley, UK). Penicillin/streptomycin, phosphate buffered salts, foetal calf serum and Minimal Essential Medium were obtained from Invitrogen (Paisley, UK). All other reagents were obtained from Sigma (Poole, UK).

### 2.2 Primary neuronal culture

Rat cerebellar granule neurons (CGNs) were dissociated from the cerebella of 7-day-old Sprague-Dawley rat pups (Harlan, Bicester, UK) and maintained as previously described (Cousin and Nicholls, 1997).

### 2.3 Fluorescence imaging

For all experiments, CGNs were plated on 25 mm coverslips at a density of ∼150,000 cells/coverslip and were immersed in incubation buffer (170 mM NaCl, 3.5 mM KCl, 0.4 mM KH_2_PO_4_, 20 mM TES (*N*-tris[hydroxy-methyl]-methyl-2-aminoethane-sulfonic acid), 5 mM NaHCO_3_, 5 mM glucose, 1.2 mM Na_2_SO_4_, 1.2 mM MgCl_2_, 1.3 mM CaCl_2_, pH 7.4). For fura-2 loading, neurons were incubated for 30 min at room temperature in incubation buffer containing 10 μM fura-2AM. After this time period fura-2 was washed off the cells with incubation buffer.

SVs were loaded with FM4-64 by stimulating CGNs for 2 min with incubation buffer containing 50 mM KCl (50 mM NaCl removed to maintain osmolarity) and 15 μM FM4-64. CGNs were washed extensively in incubation buffer and left to rest for 10 min to allow loaded SVs to recycle and become fusion competent. In dual labelling experiments, FM4-64 loading was performed after fura-2AM loading.

Loaded cells were mounted in a closed bath perfusion chamber (Warner, Hamden, CT) on the stage of a Nikon Diaphot inverted epifluorescence microscope (Tokyo, Japan) and visualized with a 20× objective lens. For the dual imaging of fura-2 and FM4-64, CGNs were sequentially excited at three wavelengths (340 and 380 nm for fura-2 and 550 nm for FM4-64) using band-pass excitation filters, a 575 nm dichroic mirror and a long-pass emission filter >575 nm (Glen Spectra, Middlesex, UK). CGNs were continuously perfused (at a rate of ∼5 ml/min) with incubation buffer during recordings and when necessary, various challenges that were designed to modulate dye fluorescence were applied by perfusion either simultaneously (50 mM KCl) or separately (0.5 M sucrose or 1 μM ionomycin). Images were acquired with a Hamamatsu Orca ER camera (Hamamatsu, Japan) and analysed offline with Simple PCI imaging software (Compix Inc., USA). Fluorescence changes in individual nerve terminals were measured by assigning regions of interest in at least 40 FM4-64 puncta (nerve terminals) per experiment and the traces were normalized to an arbitrary baseline and then averaged.

In triple imaging experiments, SYTOX Green (1 μM) was perfused onto the cells after stimulation of previously loaded fura-2/FM4-64 cells. Labelled nuclei were visualized by excitation at 485 nm with a 505 nm dichroic mirror and a 520–550 nm band-pass emission filter (Glen Spectra, UK).

The fura-2 response was calibrated by the addition of 5 mM EGTA to fura-2-loaded neurones to obtain *R*_min_, and then 10 mM CaCl_2_ plus 20 μM ionomycin to obtain *R*_max_. These values were entered into the Grynkiewicz equation (Grynkiewicz et al., 1985) to convert 340/380 ratios to [Ca^2+^]_i_ concentrations.

### 2.4 Determination of the emission spectra of fura-2

Fura-2 free pentapotassium salt at a concentration of 10 μM in incubation buffer (which contained 1.3 mM Ca^2+^, thus ensuring fura-2 was in a fully Ca^2+^-bound conformation) was excited at 340 or 380 nm in a spectrofluorimeter (Spex Fluoromax, USA) and emission readings were taken between 400 and 750 nm at 1 nm intervals. These data were plotted using Graphpad Prism (3.0) together with emission data (from 575 to 700 nm) for FM4-64 bound to CHAPS micelles excited at 550 nm (obtained from the published spectra at http://www.probes.invitrogen.com/).

## 3 Results

### 3.1 Simultaneous monitoring of [Ca^2+^]_i_ and SV turnover in single nerve terminals

**Fig. 1:**
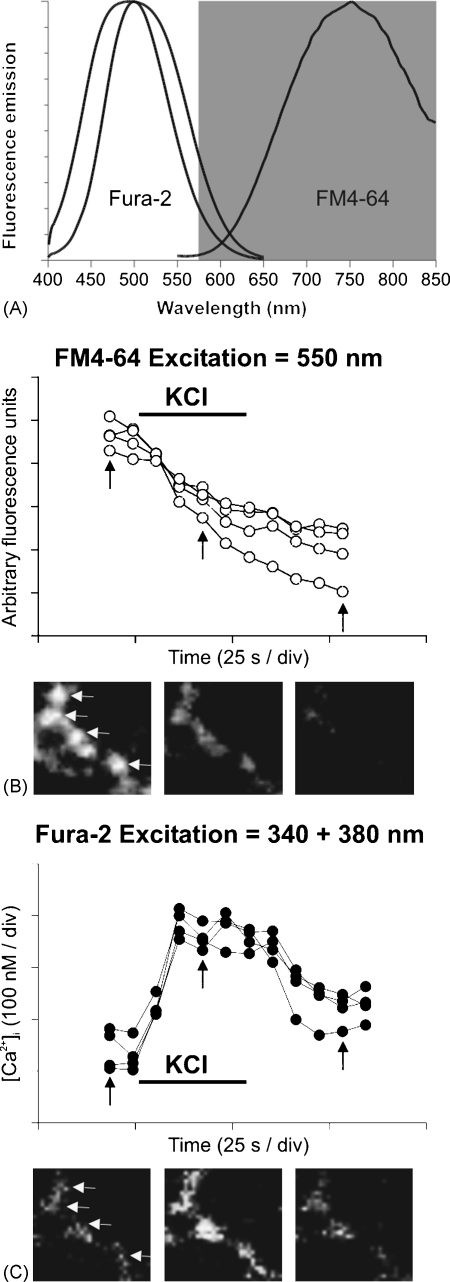
Simultaneous measurement of fura-2 and FM4-64 signals using a far red emission filter set. (A) The emission spectra of Ca^2+^-bound fura-2 excited at 340 or 380 nm shows detectable emission in the range above 575 nm (shown by shaded area). (B and C) Simultaneous imaging of FM4-64 (B) and fura-2 (C) in CGNs. CGNs were loaded with FM4-64 and fura-2 and then challenged with 50 mM KCl (bar) whilst acquiring the emitted fluorescence above 575 nm following excitation at 550 nm (B) and 340/380 nm (C). The traces represent the emission recorded at the individual nerve terminals identified in the images (white arrows). Black arrows indicate the time points of image acquisition relative to KCl stimulation, −6, 18 and 54 s. Traces are representative of five independent experiments.

An assay that simultaneously monitors both intracellular free Ca^2+^ ([Ca^2+^]_i_) and SV recycling in single nerve terminals in real time would provide new insights into the relationship between these key neuronal functions. Previous studies that have attempted this using fura-2 and FM1-43 have sometimes resulted in fluorescence bleedthrough, which complicates interpretation (Haller et al., 1999, 2001). To negate this problem we utilized the red-emitting styryl dye FM4-64 (Tan et al., 2003). However the emission spectra of fura-2 and FM4-64 are also relatively well separated (Fig. 1A). This is a problem, since to monitor neuronal responses in real time on a standard epifluorescence microscope, the same dichroic and emission filter sets have to be used. However, we noticed that while the emission maxima of fura-2 at both 340 and 380 nm excitation are approximately 500 nm, fluorescence emission continues above 575 nm (Fig. 1A). Theoretically, this should allow fura-2 emission to be monitored with a 575 nm dichroic mirror and >575 nm long pass emission filter, a configuration traditionally used with FM4-64 alone. Thus [Ca^2+^]_i_ responses and SV recycling could be monitored in real time in the same nerve terminals by exciting at 340, 380 nm (for fura-2) and 550 nm (for FM4-64) and monitoring emission at >575 nm.

As proof of principle, we tested this novel combination of dyes and emission filters by simultaneously measuring [Ca^2+^]_i_ and SV recycling in CGNs, a system which has been characterised extensively with the individual dyes (Cousin and Nicholls, 1997; Cousin et al., 1995; Tan et al., 2003). CGNs were first loaded with fura-2AM for 30 min and then with FM4-64 for 2 min using 50 mM KCl. After a rest period the loaded neurons were imaged in real time and challenged with a KCl stimulus while capturing sequential frames at either 340, 380 or 550 nm excitation. The trace in Fig. 1B depicts the fluorescence emission of the indicated nerve terminals following 550 nm excitation. As expected, depolarisation evoked a decrease in FM4-64 fluorescence, indicative of a departitioning of the dye from the SV membrane during exocytosis (Cochilla et al., 1999; Cousin and Robinson, 1999). We also observed a concomitant increase and then gradual return to baseline of the 340/380 nm emission ratio, indicating the detection of a transient [Ca^2+^]_i_ increase by fura-2. From these initial results this assay appears to monitor both [Ca^2+^]_i_ responses and SV turnover simultaneously within the same nerve terminal in real time.

### 3.2 Readout of [Ca^2+^]_i_ and SV recycling is independent of fluorescence bleedthrough

**Fig. 2:**
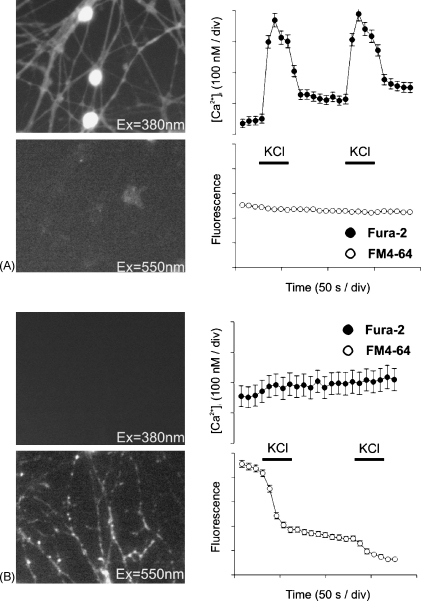
Lack of fluorescence bleedthrough between the fura-2 and FM4-64 signals. CGNs were loaded with either fura-2 (A) or FM4-64 (B) and fluorescence images recorded at 550 or 380 nm excitation (pictures on left). The right hand traces show averaged fluorescence intensities recorded at 550 nm (empty circles) or 340 and 380 nm (340/380 ratio, solid circles) from over 100 individual nerve terminals over time. The neurons were depolarised by perfusion with two consecutive 30 s pulses of 50 mM KCl at 20 and 80 s (bars). Error bars are ±S.E.M. (*n* > 100 nerve terminals). Traces are representative of at least two experiments for (A) and three experiments for (B).

To eliminate the possibility that there was any fluorescence bleedthrough, which may confound the assay readout, we performed a series of control experiments. To confirm that the excitation of fura-2 did not excite FM4-64 or vice versa, we labelled neurons with either FM4-64 or fura-2 and stimulated with 50 mM KCl. Since this stimulus evokes maximal [Ca^2+^]_i_ elevations and SV turnover in this culture system (Cousin and Nicholls, 1997; Cousin et al., 1995; Tan et al., 2003) any contamination between fluorescence signals should be observed. Fluorescence emission was recorded >575 nm during excitation at 340, 380 and 550 nm using identical exposure times to that in Fig. 1. Fig. 2A demonstrates that CGN terminals loaded with fura-2 do not emit fluorescence when excited at 550 nm apart from a small contamination by autofluorescence. The averaged traces also confirm that there is no significant change during 550 nm excitation with KCl stimulation (Fig. 2A). There was however a robust and transient increase in [Ca^2+^]_i_ recorded during 340/380 nm excitation, corresponding to depolarisation-dependent Ca^2+^ influx. Similarly, there was no significant bleedthrough in neurons loaded only with FM4-64 that were excited at either 380 nm (Fig. 2B) or 340 nm (data not shown) and there was no change in the fluorescence readout during the KCl stimulation (Fig. 2B). As expected 50 mM KCl stimulation evoked a decrease in fluorescence emission when FM4-64-loaded neurones were excited at 550 nm (Fig. 2B). Thus, there is no significant fluorescence bleedthrough in either the 340/380 nm or 550 nm excitation channels in this assay.

**Fig. 3:**
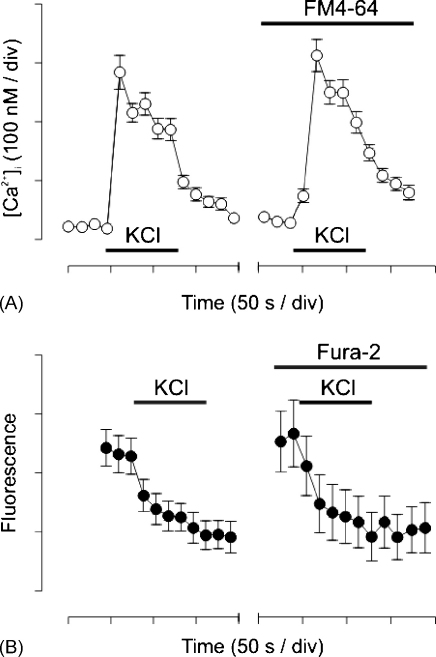
Fura-2 and FM4-64 do not interfere with either SV recycling or [Ca^2+^]_i_ responses, respectively. (A) CGNs were loaded with fura-2 and fluorescence images were recorded at 340/380 nm excitation during stimulation with 50 mM KCl (bar). After 10 min CGNs were loaded with FM4-64 and imaged in an identical manner as before. (B) CGNs were loaded with FM4-64 and fluorescence images were recorded at 550 nm excitation during stimulation with 50 mM KCl (bar). CGNs were then loaded with fura-2AM for 20 min and imaged in an identical manner as before. In both (A) and (B) the presence of either fura-2 or FM4-64 had no effect on the fluorescence output of the other. Error bars are ±S.E.M. (*n* > 20 nerve terminals). Traces are representative of at least two experiments for (A) and (B).

It is possible that fura-2 loading could have direct effects on SV recycling and conversely that FM4-64 loading could affect the evoked nerve terminal [Ca^2+^]_i_ response. To eliminate this possibility CGNs were loaded with fura-2 and the [Ca^2+^]_i_ response evoked by stimulation with 50 mM KCl was monitored using 340/380 nm excitation (Fig. 3A). Cultures were then loaded with FM4-64 and the fura-2 response to an identical stimulus was monitored again at 340/380 nm excitation (Fig. 3A). There was no significant difference between the [Ca^2+^]_i_ responses evoked before and after FM4-64 loading, indicating FM4-64 has no effect on the ability of fura-2 to report changes in [Ca^2+^]_i_. The converse was also true when the FM4-64 response to KCl stimulation was monitored at 550 nm excitation both before and after loading with fura-2AM (Fig. 3B). Thus neither fura-2 nor FM4-64 have any direct effects on each others ability to report changes in either SV recycling or [Ca^2+^]_i_.

**Fig. 4:**
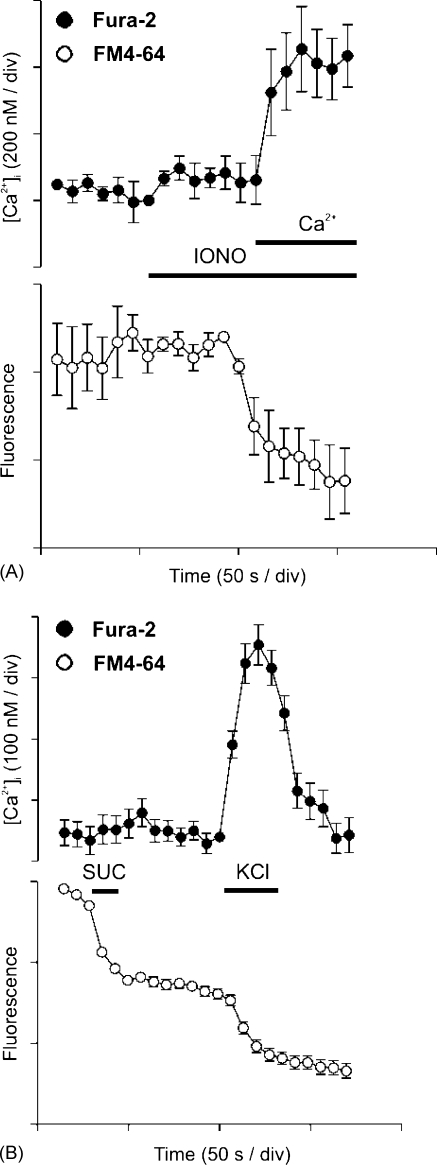
The fluorescent signals from fura-2 and FM4-64 are functionally separate. (A) CGNs loaded with FM4-64 and fura-2 were imaged in real time at 550, 340 and 380 nm excitation. Cultures were challenged with 1 μM ionomycin in the absence of CaCl_2_ (IONO bar) and then in the continuing presence of ionomycin with 1.3 mM CaCl_2_ (Ca^2+^ bar). (B) Neurons loaded and imaged as described in (A) were challenged with 500 mM sucrose for 10 s (SUC bar) and then with 50 mM KCl for 30 s (KCl bar). The traces in (A) and (B) show the average emitted fluorescence from nerve terminals above 575 nm ±S.E.M. (*n* > 100 nerve terminals) for 550 nm excitation (FM4-64, empty circles) or 340/380 nm excitation (fura-2, solid circles). Note the larger [Ca^2+^]_i_ scale in (A). Traces are representative of at least two experiments.

To demonstrate that the fluorescence signals of fura-2 and FM4-64 were functionally distinct, we used specific treatments that independently modulated either [Ca^2+^]_i_ or SV recycling. First, cultures were challenged with the Ca^2+^-ionophore ionomycin in the absence of extracellular Ca^2+^, since this treatment releases Ca^2+^ from intracellular stores but does not evoke exocytosis in CGNs (Huston et al., 1995). Ionomycin (1 μM) evoked a small but reproducible increase in the nerve terminal fura-2 signal with no effect on FM4-64 emission (Fig. 4A). Thus the [Ca^2+^]_i_ fluorescence signal is independent of the SV turnover readout. To confirm that these neurones could recycle SVs in response to stimulation, we added back extracellular Ca^2+^ (1.3 mM) in the presence of ionomycin, which caused a simultaneous increase in the fura-2 signal and corresponding decrease in the FM4-64 signal.

To determine whether the SV turnover readout was independent of the [Ca^2+^]_i_ signal, we challenged our cultures with hypertonic sucrose (500 mM), which evokes a Ca^2+^-independent fusion of release-ready SVs docked at the presynaptic plasma membrane (Rosenmund and Stevens, 1996). The sucrose challenge resulted in a rapid decrease in the FM4-64 signal, but had no effect on the fluorescence readout from fura-2 (Fig. 4B). To demonstrate that these cultures could respond to a stimulus that elevates [Ca^2+^]_i_, we applied 50 mM KCl. On application of KCl we observed a robust and transient increase in the fura-2 signal and a concomitant decrease in FM4-64 fluorescence, indicative of Ca^2+^-dependent SV exocytosis. Taken together these control experiments demonstrate that the measurement of fura-2 and FM4-64 using a 575 nm dichroic mirror and >575 nm long pass emission filter is free from bleedthrough fluorescence emission and therefore [Ca^2+^]_i_ and SV recycling can be monitored simultaneously in real time in single nerve terminals.

### 3.3 Integration of a cell viability assay

**Fig. 5:**
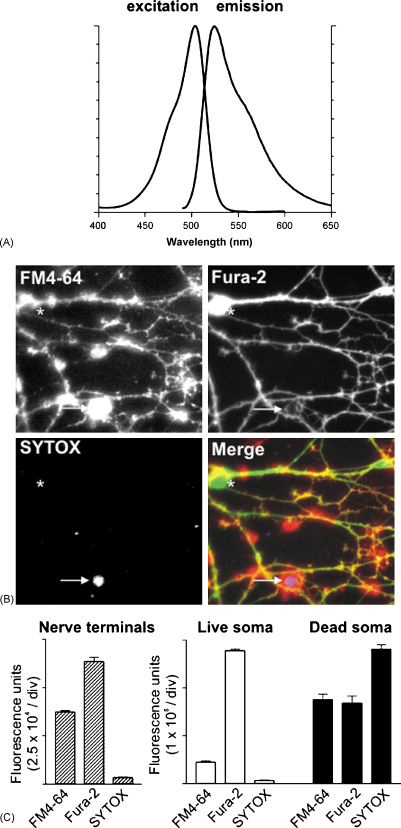
Incorporation of SYTOX Green to create an assay for three neuronal functions. (A) The excitation and emission spectra of SYTOX Green bound to DNA (http://www.probes.com). (B) CGNs were loaded with FM4-64 and fura-2 as before and images were acquired using a 575 nm dichroic at 550 nm (FM4-64) and 380 nm (fura-2) excitation. The cultures were then incubated with 1 μM SYTOX Green and images were acquired at 485 nm excitation using a 520–550 nm band pass emission filter (SYTOX). There was no detectable bleedthrough of the FM4-64 or fura-2 signals into the SYTOX image. Arrow indicates a dead cell body identified using SYTOX Green. Note the low fura-2 signal (since the plasma membrane is compromised) and the excess loading of FM4-64 (indicative of dead cell bodies). Asterisk indicates a healthy cell body with no SYTOX signal, normal fura-2 accumulation and no FM4-64 loading. A merged image is also displayed (FM4-64, red; fura-2, green; SYTOX, blue). (C) The average fluorescence intensity of triple labelled CGN nerve terminals (hatched bars), live cell bodies (open bars) and dead cell bodies (closed bars) monitored at 550 nm (FM4-64), 380 nm (fura-2AM) and 485 nm (SYTOX) is displayed ±S.E.M. (*n* = 168 nerve terminals, *n* = 122 live cell bodies, *n* = 32 dead cell bodies, data extracted from four independent experiments).

Since fura-2 and FM4-64 are excited at opposite ends of the fluorescence spectrum, we hypothesised there may be an opportunity to monitor another cellular parameter within our assay, such as cell viability. Any such reporter dye would need to have very narrow excitation and emission spectra in the fluorescein range, to ensure that there was negligible fluorescence bleedthrough into either the fura-2 or FM4-64 signals. Using these criteria we searched for candidate reporter dyes and we found the DNA binding dye SYTOX Green, which has very narrow excitation and emission spectra that peak at 500 and 530 nm, respectively (Fig. 5A). DNA-bound SYTOX Green stains the nuclei of cells with compromised plasma membranes and thus functions as an indicator of cell death.

Since 500 nm excitation also excites FM4-64 (http://www.probes.invitrogen.com/), SYTOX Green cannot be used in the same emission configuration for the simultaneous monitoring of fura-2 and FM4-64 as described above. However, the fluorescence signal should be separated by exciting at 485 nm and monitoring the resulting emission through a 520–550 nm band-pass filter (and a 505 nm dichroic mirror). This removes possible FM4-64 fluorescence bleedthrough, since FM4-64 only emits at wavelengths above 550 nm (Fig. 1A). To determine whether SYTOX Green could be employed as an additional indicator of cell viability in this assay, cultures that were previously imaged for fura-2 and FM4-64 were exposed to the dye. Fig. 5B shows a field of CGNs that have already been loaded with FM4-64 and fura-2 and then labelled and imaged for SYTOX Green. Neither FM4-64 or fura-2 staining was detectable in the SYTOX channel, indicating the dye was reporting cell death independently of [Ca^2+^]_i_ or SV recycling.

SYTOX Green only emits fluorescence when intercalated with DNA, thus no SYTOX Green fluorescence should be observed in nerve terminals. To confirm this the fluorescence intensity of triple labelled nerve terminals was monitored using the respective filter configurations for each dye. As expected no SYTOX Green fluorescence was observed in contrast to both FM4-64 and fura-2 (Fig. 5C). In addition, cell bodies that had no SYTOX Green fluorescence had strong fura-2 loading and little FM4-64 fluorescence, characteristic of viable cells (Fig. 5C). Alternatively, cell bodies that had intense SYTOX Green fluorescence had lower levels of fura-2 fluorescence and high levels of FM4-64 fluorescence, indicative of compromised neurones (Fig. 5C). The ability of SYTOX Green to accurately report cell death was confirmed by examining neurones under bright field illumination. SYTOX Green negative cell bodies had a round translucent appearance, whereas positive cell bodies appeared shrunken and dense, with apparent pycnotic nuclei (data not shown). Thus we have developed a new method to simultaneously monitor three essential neuronal processes, changes in [Ca^2+^]_i_, SV turnover and cell death.

## 4 Discussion

We have described a novel method for simultaneously measuring [Ca^2+^]_i_ and SV recycling on an epifluorescence microscope using a fixed combination of a dichroic mirror and emission filter that eliminates fluorescence bleedthrough. Furthermore, the spectral properties of the dyes employed (FM4-64 and fura-2), allow a third dye, SYTOX Green, to be applied to the same neurones to measure acute cell death. Thus we have developed an assay that provides a simultaneous and real time readout of [Ca^2+^]_i_ and SV recycling with the added value of a cell viability assay.

### 4.1 Simultaneous real time monitoring of [Ca^2+^]_i_ and SV recycling in individual nerve terminals

Previous studies have used FM1-43 in conjunction with fura-2 to monitor SV recycling and [Ca^2+^]_i_ in real time. This was achieved by exciting the dyes at 340/380 nm (fura-2) and between 470 and 490 nm (FM1-43). Some groups utilized a 400 nm dichroic mirror which reflected sufficient light (10%) above 400 nm to excite FM1-43 (Nunez et al., 2000; Shorte et al., 1995) while others used a standard 505 nm dichroic and band-pass emission filter (515–565 nm) (Burrone and Lagnado, 1997; Haller et al., 1998, 1999, 2001; Lagnado et al., 1996). When we attempted to reproduce these experiments in CGNs, we found significant fluorescence bleedthrough of FM1-43 emission into the fura-2 signal (GJOE unpublished observations). This has been observed by other groups, who were forced to lower the concentration of FM1-43 and perform back calculations to correct the fura-2 signal for bleedthrough of FM1-43 fluorescence (Haller et al., 1999, 2001). In our assay fluorescence bleedthrough has been eliminated using two modifications: (i) the use of FM4-64, which is excited in the red range of the spectrum instead of FM1-43 which is excited in the green range and (ii) measuring FM4-64 and fura-2 emission in the red range through a >575 nm long-pass emission filter. This permits a real time measurement of the fluorescence emission of these dyes by simply changing the excitation wavelength.

One of the main advantages of this assay is that [Ca^2+^]_i_ responses and SV recycling can be monitored simultaneously with the same dichroic/emission filter sets. This is an essential requirement for real time recording of these processes, since standard epifluorescence imaging systems do not have automated dichroic changers. One downside of the assay is that longer exposure times are required for fura-2 in the red range (since less fluorescence is emitted at these wavelengths) in comparison to those when monitoring emission at 500 nm, resulting in a decrease in temporal resolution. However the exposure times required to gather fura-2 emission in the red range (approximately 1 s) are well within the kinetic range to obtain physiologically relevant temporal information. One other complication may be the investigation of coloured compounds that either absorb or emit fluorescence, thus confounding the assay output. However, this problem is not unique to the assay and would affect the readout of these dyes regardless of the filter configuration.

The ability to monitor [Ca^2+^]_i_ changes and SV recycling in real time within the same nerve terminal should provide a new insight into a number of different synaptic processes that are dependent the coupling between Ca^2+^ and SV recycling. For example, it should shed new light on the behaviour of presynaptically “silent” or “mute” synapses, a phenomenon where nerve terminal stimulation elicits no SV recycling response until a certain “unsilencing” stimulus is applied (Voronin and Cherubini, 2004). This assay will allow a dissection of the effect of the unsilencing stimulus to be monitored on both the [Ca^2+^]_i_ response and SV recycling in real time. In addition more detailed investigations of the role of synaptic signalling pathways and post-translational modifications will be possible using this assay. For example, it will be possible to visualize the effect of signalling cascades on the [Ca^2+^]_i_ response and SV recycling simultaneously to determine whether their effect is upstream or downstream of Ca^2+^ influx. It should now also be possible to directly relate effects on SV recycling evoked by different stimulation frequencies to the Ca^2+^ channel subtypes present within the same nerve terminal. Finally, the relationship between calcium influx and the mode of SV recycling can be investigated by employing red emitting styryl dyes of different hydrophobicity such as FM5-95. FM5-95 is more hydrophilic than FM4-64 and thus departitions from membranes more quickly. A similar relationship between the green emitting dyes FM2-10 and FM1-43 has allowed researchers to infer effects on either the length of time SVs spend fused at the plasma membrane (Klingauf et al., 1998) or the mode of SV endocytosis (Richards et al., 2000; Virmani et al., 2003). Thus the [Ca^2+^]_i_ and SV recycling response to different patterns of physiological stimuli could be simultaneously monitored to gain novel insights into the molecular mechanism of these events.

### 4.2 Applications outwith SV recycling

The technology applied in this assay is not restricted to studies of SV recycling and its relationship to [Ca^2+^]_i_. It will also allow the simultaneous monitoring of many other essential cellular functions in both neuronal and non-neuronal cells. This is because the filter sets employed allow the use of any reporter dye that emits in the red range, since they are already optimised for this signal. For example FM4-64 could be substituted for dyes that report either plasma membrane lipid hydrolysis (BODIPY 558/568); plasma membrane potential (Di-8-ANNEPS); movement of acidic organelles (Lysotracker Red); mitochondrial membrane potential (Rhodamine 123/TMRM) or superoxide production (MitoSOX Red). Similarly fura-2 could be substituted for its derivates SBFI or PBFI to monitor [Na^+^]_i_ or [K^+^]_i_, respectively, although the low quantum yield of these UV dyes may restrict their use in the far red range. All of the above parameters will be of great interest to researchers since they are key targets for drugs to combat cancer (lipid hydrolysis), apoptosis (mitochondrial membrane potential), neurodegenerative diseases (superoxide production), drug delivery (movement of acidic organelles) and disorders of cellular excitability such as epilepsy (plasma membrane potential). Furthermore, the incorporation of a cell death assay using SYTOX Green adds considerable value to all of these studies since it allows cell death to be temporally related to other key intracellular events. In this paper SYTOX Green is added post stimulation, but we see no reason why it could not be added during the fura-2 loading period, since it will not be excited by either fura-2 (340 or 380 nm) or FM4-64 (550 nm) wavelengths. This would allow cell viability to be assessed both before, during and after initiation of the experiment adding further value to the assay.

### 4.3 A multiplexed assay of three essential neuronal parameters

The incorporation of a cell death assay into the existing [Ca^2+^]_i_ and SV recycling assay means that we have developed a method for measuring three essential neuronal properties within the same cell. The development of such multiplexed assays that simultaneously monitor multiple cellular events has resulted in the new field of high content screening (HCS) (Abraham et al., 2004). We propose that this assay is an ideal candidate for translation into a HCS format and would be an excellent method for screening the effect of small molecule inhibitors on neuronal function in a cellular context. Importantly, there are no HCS assays currently available that examine these three key neuronal parameters, making this assay very attractive for drug screening studies that target diseases of cell excitability and/or SV recycling such as epilepsy, schizophrenia, bipolar disorder and depression.

In conclusion we report an assay that simultaneously monitors both [Ca^2+^]_i_ responses and SV recycling in real time in the same nerve terminal. This has the potential to address previously answered questions regarding the coupling of Ca^2+^ influx to neurotransmitter release. We have added an extra parameter to the assay through use of a cell viability indicator and we propose that this assay is an ideal candidate for translation into a HCS format.

## References

[bib1] Abraham V.C., Taylor D.L., Haskins J.R. (2004). High content screening applied to large-scale cell biology. Trends Biotechnol.

[bib2] Betz W.J., Bewick G.S. (1992). Optical analysis of synaptic vesicle recycling at the frog neuromuscular junction. Science.

[bib3] Burrone J., Lagnado L. (1997). Electrical resonance and Ca^2+^ influx in the synaptic terminal of depolarising bipolar cells from the goldfish retina. J Physiol.

[bib4] Cochilla A.J., Angleson J.K., Betz W.J. (1999). Monitoring secretory membrane with FM1-43 fluorescence. Annu Rev Neurosci.

[bib5] Cousin M.A., Nicholls D.G. (1997). Synaptic vesicle recycling in cultured cerebellar granule cells: role of vesicular acidification and refilling. J Neurochem.

[bib6] Cousin M.A., Robinson P.J. (1999). Mechanisms of synaptic vesicle recycling illuminated by fluorescent dyes. J Neurochem.

[bib7] Cousin M.A., Held B., Nicholls D.G. (1995). Exocytosis and selective neurite calcium responses in rat cerebellar granule cells during field stimulation. Eur J Neurosci.

[bib8] Dunlap K., Luebke J.I., Turner T.J. (1995). Exocytotic Ca^2+^ channels in mammalian central neurons. Trends Neurosci.

[bib9] Grynkiewicz G., Poenie M., Tsien R.Y. (1985). A new generation of Ca^2+^ indicators with greatly improved fluorescence properties. J Biol Chem.

[bib10] Haller T., Ortmayr J., Friedrich F., Volkl H., Dietl P. (1998). Dynamics of surfactant release in alveolar type II cells. Proc Natl Acad Sci USA.

[bib11] Haller T., Auktor K., Frick M., Mair N., Dietl P. (1999). Threshold calcium levels for lamellar body exocytosis in type II pneumocytes. Am J Physiol.

[bib12] Haller T., Dietl P., Pfaller K., Frick M., Mair N., Paulmichl M., Hess M.W., Furst J., Maly K. (2001). Fusion pore expansion is a slow, discontinuous, and Ca^2+^-dependent process regulating secretion from alveolar type II cells. J Cell Biol.

[bib13] Hell J.W., Westenbroek R.E., Elliott E.M., Catterall W.A. (1994). Differential phosphorylation, localization, and function of distinct alpha 1 subunits of neuronal calcium channels. Two size forms for class B, C, and D alpha 1 subunits with different COOH-termini. Ann NY Acad Sci.

[bib14] Huston E., Cullen G.P., Burley J.R., Dolphin A.C. (1995). The involvement of multiple calcium channel sub-types in glutamate release from cerebellar granule cells and its modulation by GABAB receptor activation. Neuroscience.

[bib15] Klingauf J., Kavalali E.T., Tsien R.W. (1998). Kinetics and regulation of fast endocytosis at hippocampal synapses. Nature.

[bib16] Lagnado L., Gomis A., Job C. (1996). Continuous vesicle cycling in the synaptic terminal of retinal bipolar cells. Neuron.

[bib17] Matthews G. (1996). Synaptic exocytosis and endocytosis: capacitance measurements. Curr Opin Neurobiol.

[bib18] Murthy V.N., De Camilli P. (2003). Cell biology of the presynaptic terminal. Annu Rev Neurosci.

[bib19] Murthy V.N., Sejnowski T.J., Stevens C.F. (1997). Heterogeneous release properties of visualized individual hippocampal synapses. Neuron.

[bib20] Nicholls D.G. (1993). The glutamatergic nerve terminal. Eur J Biochem.

[bib21] Nunez L., Villalobos C., Boockfor F.R., Frawley L.S. (2000). The relationship between pulsatile secretion and calcium dynamics in single, living gonadotropin-releasing hormone neurons. Endocrinology.

[bib22] Passafaro M., Clementi F., Pollo A., Carbone E., Sher E. (1994). Omega-conotoxin and Cd^2+^ stimulate the recruitment to the plasmamembrane of an intracellular pool of voltage-operated Ca^2+^ channels. Neuron.

[bib23] Passafaro M., Rosa P., Sala C., Clementi F., Sher E. (1996). N-type Ca^2+^ channels are present in secretory granules and are transiently translocated to the plasma membrane during regulated exocytosis. J Biol Chem.

[bib24] Richards D.A., Guatimosim C., Betz W.J. (2000). Two endocytic recycling routes selectively fill two vesicle pools in frog motor nerve terminals. Neuron.

[bib25] Rosenmund C., Stevens C.F. (1996). Definition of the readily releasable pool of vesicles at hippocampal synapses. Neuron.

[bib26] Ryan T.A., Reuter H., Wendland B., Schweizer F.E., Tsien R.W., Smith S.J. (1993). The kinetics of synaptic vesicle recycling measured at single presynaptic boutons. Neuron.

[bib27] Shorte S.L., Stafford S.J., Collett V.J., Schofield J.G. (1995). Simultaneous measurement of [Ca^2+^]_i_ and secretion-coupled membrane turnover, by single cell fluorescence microscopy. Cell Calcium.

[bib28] Spafford J.D., Zamponi G.W. (2003). Functional interactions between presynaptic calcium channels and the neurotransmitter release machinery. Curr Opin Neurobiol.

[bib29] Tan T.C., Valova V.A., Malladi C.S., Graham M.E., Berven L.A., Jupp O.J., Hansra G., McClure S.J., Sarcevic B., Boadle R.A., Larsen M.R., Cousin M.A., Robinson P.J. (2003). Cdk5 is essential for synaptic vesicle endocytosis. Nat Cell Biol.

[bib30] Virmani T., Han W., Liu X., Sudhof T.C., Kavalali E.T. (2003). Synaptotagmin 7 splice variants differentially regulate synaptic vesicle recycling. EMBO J.

[bib31] Voronin L.L., Cherubini E. (2004). ‘Deaf, mute and whispering’ silent synapses: their role in synaptic plasticity. J Physiol.

